# Deprescribing, shared decision-making, and older people: perspectives in primary care

**DOI:** 10.1186/s40545-023-00671-9

**Published:** 2023-11-27

**Authors:** Sumaiah Alrawiai

**Affiliations:** Department of Health Information Management and Technology, College of Public Health, Imam Abdulrahman Bin Faisal University, 34212 Dammam, Saudi Arabia

**Keywords:** Shared decision-making, Deprescribing, Polypharmacy

## Abstract

Polypharmacy is an issue that affects many people, especially older adults, and could result in negative outcomes such as lower medication adherence and an increase in the likelihood of adverse drug reactions. Deprescribing is a possible solution to mitigating this issue. Examining polypharmacy and deprescribing in primary care settings is important as it could help older adults living in the community and their relatives by lowering their treatment burden and medication cost. Some guidelines have been developed to help with the deprescribing process; however, these guidelines are not applicable to all patients and situations. Thus, the deprescribing process needs to be based mainly on the patient’s current situations, preferences, and values and this could be achieved using shared decision-making. However, some barriers slow down the process to deprescribe in primary care settings and measures should be taken to overcome these barriers. This review aims to examine the current situation of deprescribing, especially in primary care settings, and how SDM can be used to optimize the deprescribing process. To achieve this an illustration using one prominent model in SDM and one prominent model in deprescribing will be presented to showcase how SDM can be used in the deprescribing process.

## Introduction

Many older adults have comorbidities and need to use multiple medications to treat their current conditions and prevent future illnesses [1]. Polypharmacy, which can be defined as the regular use of five or more medications, is a common issue among older people [2, 3]. Polypharmacy is not inherently bad; however, if the medications taken by the patient are unnecessary or could cause harm that outweighs the benefits, then the medication list needs to be reviewed with the patient. Because there is a possibility of drug–drug and/or drug–disease interactions, polypharmacy can increase the risk of adverse drug reactions, hospitalizations, falls, and deaths [4–7]. It can also lead to lower adherence, increased medication burden, and an increase in avoidable treatment costs [3, 8, 9]. It has been shown that the addition of as much as one drug considerably increases the likelihood of an adverse drug reaction [10].

In addition to the fact that older people have comorbidities requiring several medications, the trend to prescribe preventive medicine can also contribute to an increase in the number of medications they have to take [11]. Compounding the problem is the fact that many guidelines are developed for a single disease; thus, a new treatment recommended for the patient may fail to detail how it might affect and interact with other medications [5]. Further, older people with comorbidities who are taking more than one drug are usually underrepresented in the trials for these drugs and in the development of the guidelines, leading to a failure to identify possible issues that could potentially increase the risks for this particular population [12, 13]. Thus, it is essential, especially for older adults, to examine the appropriateness of medications for each individual patient [14, 15], in conjunction with the evidence-based guidelines.

It is estimated that more than a third of older adults in primary care have been prescribed potentially inappropriate medications [16]. Deprescribing could be one of the best solutions to the polypharmacy issue, that is, tailoring the medication to the individual’s current needs and preferences. Studies show evidence of deprescribing’s efficiency in mitigating the risks associated with polypharmacy [17, 18].

Although a number of reviews existed on the importance of SDM as part of the deprescribing process, in this review the using of one model of SDM and how it can be employed within a deprescribing model could shed light on the possibility of using established models of SDM and match their elements with corresponding steps in deprescribing models.

### What is deprescribing?

Deprescribing is defined as the “systematic process of identifying and discontinuing drugs in instances in which existing or potential harms outweigh existing or potential benefits within the context of an individual patient’s care goals, current level of functioning, life expectancy, values, and preferences” [6].

Deprescribing is a highly personalized process in which each patient is viewed as a unique individual with their own issues, preferences, and number of medications.

Medications can be divided into two broad categories: those used for preventive purposes and those used for symptom alleviation and treatment purposes. Because both types of medication have the potential to be deprescribed, this division could help with the decision of which drug to stop [6]. The division might also help in the discussion of deprescribing options with the patient, because doctors can explain each drug’s purpose and category [5]. Some have suggested deprescribing preventive medicine first, especially considering that some of these medications require a long period before the patient can see any benefits; older adults with life-limiting illnesses would be unlikely to reap the benefits, considering their life expectancy [19]. Other factors that would indicate a need to initiate the deprescribing process include the following: patient’s wishes; adverse drug reaction; decrease in patient’s mobility and increase in the risk of falls; and effects on patient’s mood and cognition, for example, through drugs that could increase the possibility of depression or lead to delirium [20–22]. Studies show that deprescribing could result in many positive outcomes [23, 24]. A systematic review and meta-analysis study found that deprescribing interventions in nursing homes resulted in a significant decrease in the number of patients with potentially inappropriate medications. Further, the review found that interventions employing medication review led to a reduction in all-cause mortality and fallers [25].

### Polypharmacy in primary care

The issue of polypharmacy is more common than expected in primary care, and it is not exclusive to hospitals and nursing homes. A study using meta-analysis to examine the prevalence of potentially inappropriate prescribing for older people in primary care settings found a prevalence of 33.3% [16]. This is considerably higher than what might be expected in community settings. Reasons for this number can be attributed to a variety of factors; for example, an increasing number of older people are having their chronic diseases managed in primary care settings, and many members of this population have comorbidities. This will likely require them to take more than one medication to treat the different conditions. It should be noted here that this meta-analysis mostly includes studies from high-income countries, and thus the results may not accurately represent the prevalence of potentially inappropriate prescribing in the world in general. However, the meta-analysis provides much-needed information on whether the primary care setting is affected by the issue of inappropriate prescribing, as is known to be the case in tertiary hospitals and nursing homes. Further, the study shows a need to conduct more research on the prevalence of inappropriate prescribing in other countries to gain a full picture of its impact. Although fewer studies have been conducted on the topic in developing countries, these studies show that the issue of inappropriate prescribing is prevalent in developing countries as well [26, 27].

In primary care settings, general practitioners (GPs) can be viewed as the ideal health professionals to lead the deprescribing efforts, for a number of reasons: they are the first point of contact with the patient; most of them have built a trusting relationship with their patients; they are increasingly becoming those responsible for following up on the management of chronic conditions; and they are responsible for care coordination in many healthcare systems around the world [28, 29]. They know the patients’ medical history and what medications they are currently taking and previously have used.

To assist healthcare professionals (HCPs) in the deprescribing process, a number of deprescribing tools have been developed, such as the Beers criteria [30], STOPP/START criteria [31], and other explicit-criteria tools to be used for deprescribing. Nevertheless, in many cases the use of these tools is not applicable, and implicit criteria that are usually based on the HCP’s judgment can be used for the deprescribing process [28].

A number of studies show that HCPs are reluctant to start the deprescribing process, for a number of reasons. For example, HCPs worry that their patients might interpret the discussion of such a process as “giving up on them” [32, 33]. However, patients do not necessarily have this thought when presented with the choice to deprescribe a medication, as shown in a study by Kua et al. [34]. In this study, patients’ and caregivers’ views towards deprescribing were examined in Singapore by using a survey. It was found that most patients seemed to view the deprescribing process in a more positive light, not feeling that HCPs are giving up on them when suggesting deprescribing a medication.

These results indicate a need to understand patients’ values and perspectives without making unsupported assumptions based on a subgroup of patients. Shared decision-making can ensure that the patient is involved and is an active participant in the deprescribing process.

### Shared decision-making (SDM) and deprescribing

When starting the process of deprescribing, it is essential to discuss with the patients their overall goals and objectives in relation to their health and general wellbeing. Do their main goals aim to improve their mobility and mood, which a certain medication might be affecting? Or do they want to live longer even at the cost of a lower quality of life? When initiating a deprescribing process, understanding the patients’ perspectives is paramount because it can affect whether the patients welcome efforts made to help them lower their medication load or else view such efforts negatively.

In a qualitative study in which older people and their companions were interviewed, three types of attitudes towards medications and deprescribing were identified [35]. One group of older people was resistant or had negative views of deprescribing; they were attached to their medications and viewed them as essential for their health and wellbeing. Others were more open and wanted to consider the option to deprescribe medications, and they were interested in being part of the decision-making process. The last group was more passive and had no awareness of deprescribing as an option, and if there was a decision to be made, they preferred to defer it to their doctors [35]. This study shows the diversity of older people and their differing attitudes towards deprescribing, which dispels the myth that older adults are a homogenous group. Further, the study highlights the importance not only of communicating with the patients to accurately understand their attitudes towards deprescribing but also of devising strategies of how to approach the subject of deprescribing without making any assumptions about them but rather inviting them to share their preferences toward participation in decision-making and their views toward deprescribing.

Shared decision-making (SDM) is essential to the deprescribing process, as evidenced by many studies, and it is a central element of person-centered care [11, 36, 37]. To illustrate how SDM can assist in the deprescribing process, both Elwyn et al.’s [38] modified three-talk model and Scott et al.’s [6] deprescribing process are used in this review. A step from the Elwyn et al.’s model, the Option Talk, is used to showcase the employment of SDM in the deprescribing process, and it is matched with the corresponding steps from Scott et al.’s deprescribing protocol.

The three-talk model consists of three steps, with the possibility of moving back and forth between them: the Team Talk, Option Talk, and Decision Talk [38]. Each step describes fundamental elements of the decision-making process. To aid HCPs in deprescribing medications, Scott et al. [6] developed a deprescribing protocol that consists of five steps. As stated earlier, these two models are used in this review to show how SDM can be implemented during a deprescribing process.

Because the Option Talk of Elwyn et al.’s model refers to the discussion with the patient of the different options available and uses risk communication [38], it seems that the Option Talk can be used in step 3 and step 4 of the deprescribing model: “Assess each drug for its eligibility to be discontinued” and “Prioritize drugs for discontinuation”, respectively.

At first glance, step 3 of the deprescribing model, “Assess each drug for its eligibility to be discontinued”, might not seem like a fit for the Option Talk, considering that the aim of this step is to decide which medications might be deprescribed. However, if one looks at the tasks under this step, it becomes clear that using the Option Talk is needed here. For example, HCPs cannot decide whether a drug is “imposing an unacceptable treatment burden”. Answering the question necessitates eliciting patients’ views on whether there is a burden or not; what might be burdensome for one patient might not be viewed as an issue by another. As such, communicating with the patient and inviting them to voice issues regarding their medications cannot be overstated and are indispensable to achieving the aim of step 3.

Step 4, “Prioritize drugs for discontinuation”, focuses on discussing different options regarding which drug, if any, to discontinue. To this end, the HCPs need to inform the patient both of the medications that can be considered for deprescribing and of the possible consequences of attempting the cessation of these medications. Communication of the possible risks and benefits should be done in a way that the patient can easily understand, so they can arrive at an informed choice. There are a number of recommendations for how to communicate options to patients [39, 40]. Based on a review of the literature, Fagerlin et al. [41] suggest 10 ways that can help clearly convey the risks and benefits to the patients. The first three recommendations are supported by the strongest evidence, according to the authors. The first recommendation is to use “plain language” that can be easily understood by patients. Second, because relative risk can present a disproportional picture, using absolute risk when communicating statistical information is preferable. The third recommendation suggests the use of pictographs when communicating risks and benefits to patients [41].

When the medications for deprescribing are being prioritized, the risks and benefits of each medication-deprescribing option need to be explained clearly to the patients, including possible benefits resulting from deprescribing and potential withdrawal effects or relapses. This issue is particularly important to keep in mind when dealing with older adults, who might have age-related cognitive issues that may affect both how they comprehend information and how they view and weigh information. For example, they might give greater weight to positive information or have difficulty considering many options [11].

### Barriers to deprescribing in primary care

One of the main barriers to deprescribing in primary care settings is the GP’s reluctance to initiate deprescribing without a prior issue, such as an adverse drug reaction. This reluctance may stem from uncertainty about what could result from deprescribing certain medications, including possible side effects and the reemergence of symptoms. The lack of guidelines detailing the process of deprescribing, especially for patients with comorbidities, contributes to this uncertainty [32, 33]. GPs also cite their hesitancy to deprescribe a medication if it has been prescribed by a specialist at the hospital [42]. Possible barriers reported by primary care doctors are legal action, patients’ complaints, and a general lack of confidence to deprescribe due to insufficient training [32]. Lack of time is also an issue for GPs; considering the complexity of the deprescribing process, doctors might be understandably reluctant, without a pressing need, to initiate such a process with their patients [33, 42]. Finally, fear of withdrawal effects is an issue shared by both doctors and patients when it comes to deprescribing [42].

In line with primary care doctors’ reluctance to deprescribe a medication prescribed by a specialist, a study that surveyed patients regarding their acceptance of deprescribing based on the deprescriber’s and prescriber’s qualifications shows that there is a basis for such a worry [43]: of the respondents, 38% answered that they would not like the primary care doctors to deprescribe a medication that was prescribed by a specialist [43]. This indicates a barrier to deprescribing from a patient’s perspective. However, in a qualitative study with older adults and their carers, participants reported that their valuation of a prescription is based on the relationship they have with their providers rather than the providers’ qualifications [44]. This indicates a need for further examination of this area to understand how it could potentially affect patients’ responsiveness to the deprescribing process and reinforce the importance of building a trusting relationship with the patient. As for HCPs hesitations to deprescribe out of fear that deprescribing will lead to negative outcomes or reemergence of symptoms, a study showed that out of 704 medicines stopped in 298 patients, only nine adverse effects were observed, and none of them were serious and have been corrected with either restarting of the medications, giving an alternative, or monitoring the patient [37]. These studies should mitigate HCPs’ fears that patients might respond negatively to deprescribing.

Systematic reviews that examined the barriers and facilitators to deprescribing in primary care settings presented many possible barriers and facilitators from patients and HCPs perspectives. Facilitators include having a trusting relationship between HCPs and patients; employing SDM tools; prudent prescribing; involving HCPs in the designing of the deprescribing intervention; provision of deprescribing resources; and involvement of pharmacists in the deprescribing process [33, 45].

## Conclusion

In conclusion, polypharmacy is a serious issue that affects many older adults and leads to many negative outcomes, including a lower quality of life. Deprescribing is one possible solution to this issue that showed positive outcomes, and for it to succeed effectively, adopting SDM is imperative. Many barriers hinder the deprescribing process, and efforts should be made to mitigate the effects of these barriers. This includes the development of guidelines for deprescribing, employing a more collaborative approach by involving pharmacists in the process of deprescribing, and building a trusting relationship with the patient and involve them in the deprescribing process.

## Data Availability

Not applicable.
